# The Effect of Cochlear Implantation on the Improvement of the Auditory Performance in 2-7 Years old Children, Shiraz 2004-2008

**DOI:** 10.5812/ircmj.4033

**Published:** 2013-03-05

**Authors:** Sayed Basir Hashemi, Abdolreza Rajaeefard, Hasan Norouzpour, Hamid Reza Tabatabaee, Leila Monshizadeh

**Affiliations:** 1 ENT Department, Shiraz University of Medical Sciences, Shiraz, IR Iran; 2 Epidemiology Department, Shiraz University of Medical Sciences, Shiraz, IR Iran; 3 Fars Cochlear Implantation Center, Shiraz, IR Iran

**Keywords:** Cochlear Implantation, Hearing

## Abstract

**Background:**

Hearing loss is the most common sensorineural deficiency in human beings. Cochlear implantation is introduced worldwide to treat the severe to profound sensorineural hearing loss, and can result in both speech comprehension and production.

**Objectives:**

The present study aims to determine the effect of cochlear implantation on the improvement of the auditory performance in 2-7 years old children.

**Patients and Methods:**

The present follow-up study is a kind of cohort study which was conducted on 98 children between 2-7 years old who had referred to Fars Cochlear Implantation Center. The patients’ information was gathered from their profiles both before and after the operation. The auditory performance score was obtained in 3 stages; 6 months, 1 year, and 2 years after the cochlear implantation through the Cap test. The data was analyzed using the nonparametric Friedman test as well as Mann-Withney, Kruskal-Wallis, and Spearman's Ranks Correlation coefficients.

**Results:**

The mean and the median of the auditory performance score of the children who had undergone the cochlear implantation revealed a significant improvement from 6 months to 1 year, and 2 years after the implantation. It showed a significant statistical association between implantation age, type of hearing loss, regular reference, and the length of being present in the rehabilitation program with the auditory performance. It showed no significant association between sex, mother’s level of education, being monolingual or bilingual, and family size with the auditory performance.

**Conclusions:**

This study revealed that the type of hearing loss, Presence in the rehabilitation program, and the age of cochlear implantation can be major prognostic factors of the response to the treatment, then the country’s health policy makers and health planners must executively take into account the infants’ hearing screening program during the first 6 month of age.

## 1. Background

Hearing loss – or hearing disorder – is the most common sensorineural deficiency in human beings (1-3). Hearing deficiency is defined as an abnormal or a reduced auditory performance which occurs due to some hearing disorders (2). All societies are involved with hearing loss. Moreover, this disorder highly affects the people’s both individual and social life (3). Humans first learn the language which is spoken in the environment, and then start to speak. In other words, if an individual is not able to hear, he or she would not learn to speak correctly (4). In the U.S., hearing loss is the most common communication disorder; in a way that the number of patients having hearing loss is more than the total number of patients with heart diseases, cerebral palsy, epilepsy, blindness, and multiple sclerosis (1). The largest proportion of hearing deficiencies goes to Asia with 2.6 in 1000 live birth with hearing disorders every year. It is estimated that 4000 newborn infants with hearing loss are annually born in Iran; nevertheless, no definite statistics exist in this regard (5). Other statistics have revealed that, on the average, 26% of middle-aged, most individuals over 65, and almost all individuals over 80 have some kinds of hearing loss (3). The major causes of hearing disorders are genetic factors, and environmental factors which include health, economic, social, educational, as well as cultural status, and particularly, consanguineous marriages. Overall, hearing disorders in prelingual categorized into two groups of congenital and acquired disorders (1). Consanguineous marriages, and mothers being infected by measles, herpes, cytomegalovirus, syphilis, toxoplasmosis, or using drugs in pregnancy can be considered as congenital factors. On the other hand, being infected by diseases such as neonatal jaundice, Orion, meningitis, and upper respiratory tract infections, experience of accident and stroke, low weight at the time of birth, and noise are considered as acquired factors which occur after birth (1, 3, 5). Nevertheless, some studies believe middle ear infections to be the most important factor (3). Hearing loss is classified into 4 groups of mild (hearing fall is 25-40db), moderate (40-70db), severe (70-90db), and profound (more than90db); and individuals are not able to hear in severe and profound hearing loss (2). Cochlear implantation, which is a major standard medical as well as engineering achievement, can be used as the final treatment for the children with severe and profound bilateral hearing loss who cannot be treated by hearing aids, and sound reinforcing instruments. This method is of great help in speech comprehension and production, in the children (6, 7). Cochlear implantation is introduced as the treatment of severe to profound sensorineural hearing loss around the world (8). Since hearing is restored after the implantation, speech is also expected to improve, and eventually, individuals are able to adjust their speech characteristics, such as the volume of their voice, and the tone of their speech, based on the voice they produce (4). Cochlear implantation is performed through the stages of studying and evaluation, operation and cochlear implantation, and finally, speech processor planning and rehabilitation (4). Previous studies revealed that cochlear implantation in lower ages reduces the hearing deprivation, and at the same time, improves both hearing and speaking (9, 10).

## 2. Objectives

Therefore, the present study aims to determine the effectiveness of cochlear implantation on the improvement of the auditory performance in 2-7 years old prelingual (inclusion criteria) children in Fars Cochlear Implantation Center.

## 3. Patients and Methods

The present follow-up study is a kind of cohort study which was conducted on 105 children between 2 and 7 years old who had undergone operations in Fars Cochlear Implantation Center, Khalili hospital, Shiraz, Iran – which is the only center in Southern Iran – from 2004 to 2008. The inclusion criteria of the study were permanent deafness before the verbal development ( prelingual) as well as other disabilities (n = 102). Four patients who failed to be followed-up for 3 months after the implantation were excluded from the study. Finally, 98 patients’ information was gathered from their profiles both before and after the operation. Moreover, if necessary, the patients were contacted using the recorded addresses, and phone numbers to complete the data. The data was gathered through the questionnaires in the patients’ profiles which had been completed by the center’s staff before the operation, and at the first admission. The data related to the patients’ auditory performance was recorded in their postoperation profiles, and was matched with the Cap test by a speech therapist. This test – as the most important scale for measuring the children’s auditory performance after the cochlear implantation – can be used even for very young children, and also, is both repeatable and reliable. Moreover, it is different from other methods of evaluation since it can be easily comprehended, and conducted even by families and nonspecialists. The Cap test includes 8 hearing scales ranging from not being aware of the environmental sounds, to use the telephone with familiar individuals (11).

### 3.1. Statistical Analysis

The auditory performance score obtained in 3 stages of 6 months, 1 year, and 2 years after the cochlear implantation was considered as the dependent variable. The gathered data was analyzed through the SPSS statistical software (version 11.5). At first, the mean of the auditory performance scores obtained 6, 12, and 24 months after the implantation was calculated. Then, the Kolmogorov-Smirnov test was used to determine the normal distribution of the data. However, since the data did not follow the normal distribution, nonparametric tests were used for comparison. In fact, regarding the dependability of the individuals’ data, the Friedman nonparametric test together with Wilcoxon test was used to compare the difference between the auditory performances scores obtained in the 3 stages. Moreover, according to the variables scale as well as the classification of the independent qualitative variables, the statistical tests of Mann-Withney U, Kruskal-Wallis H, and also Spearman Ranks Correlation Coefficients were used to determine the association between the independent variables and the dependent variables. Besides, P < 0.05 was considered as statistically significant.

## 4. Results

The mean age at the time of the implantation was 41.77 + 13.1 months ranging from 24 to 84. Regarding sex, 50% of the children were male who had been randomly selected. The mean number of times the patients were present in the rehabilitation program in 6 months, 1 year, and 2 years after the implantation were 32.2 + 10, 56.63 + 18.1, and 60.4 + 19.7 respectively. Furthermore, the mean and the median of the auditory performance of the children who had undergone the cochlear implantation were computed for 6 months, 1 year, and 2 years after the operation (Table 1). The results revealed a significant difference between them (P < 0.05). Also, the paired comparison, which was conducted, revealed that all the groups were different (P < 0.001). Studying the association between the variables, which was performed in univariate analysis, revealed a statistically strong association between the independent variables of the type of hearing loss, and the regular presence in as well as and the number of the references to the rehabilitation program, and the dependent variables of the auditory performance scores obtained in the 3 stages (Table 3). However, no significant association was found between the independent variables of sex, mother’s level of education, the families’ hearing loss status, the age difference between the patients, and his or her previous or next child, being monolingual or bilingual, and family size, and the dependent variables of the auditory performance scores obtained in the 3 stages (Table 2 and 3). Moreover, no significant association was observed between the mother’s occupation and the auditory performance scores obtained for 6 and 12 months after the implantation; however, the association revealed to be significant 24 months after the implantation (Table 2). The present study also revealed a highly significant association between the age at the time of implantation and the auditory performance scores obtained for12 and 24 months after the operation, while this association was not significant 6 months after the implantation (Table 3). In the present study, the results of the correlation analysis revealed a weak positive association between the auditory performance scores obtained 2 years after the implantation, and the length of the presence in the rehabilitation program (P < 0.02, r = 0.22). The results also revealed a significant positive linear association between the auditory performance scores obtained 6 months after the operation, and the length of time an individual was present in the rehabilitation program (P < 0.02, r = 0.23). A significant positive association was also observed between the number of the times an individual was present in the rehabilitation program during 12 and 24 months, and the auditory performance scores obtained in each stage (P < 0.001, r = 0.36).

**Table 1.** The children’s Auditory Performance Median, and Mean Scores Obtained in 6, 12, and 24 Months After the Implantation (n = 98)

The Auditory Performance Mean Score	Mean ± SD	Median (minimum-maximum)	P value
**6 Months after the operation**	2.8 ± 1.03	3(0-5)	< 0.001
**12 Months after the operation**	4.36 ± 1.04	5(1-7)	
**24 Months after the operation**	5.34 ± 1.02	5(2-7)	

**Table 2.** The Association Between the Independent Demographic Variables, and the Auditory Performance Scores Obtained 6, 12, and 24 Months After the Implantation (n = 98)

Variable	The Auditory Performance Graph					
	The median of the score in 6 months		The median of the score in 12 months		The median of the score in 24 months	
	Median (q1-q3)	P value ^a^	Median (q1-q3)	P value ^a^	Median (q1-q3)	P value ^a^
**Sex**		0.26		0.77		0.29
Male	3(2-4)		5(3-5)		5(4-5)	
Female	3(2-3)		5(4-6)		5(5-6)	
**Mother's level of education**		0.53		0.17		0.057
Illiterate	3(2-3)		5(3-4)		5(4-5)	
School education	3(2-4)		5(4-5)		5(5-6)	
Diploma and higher education	3(2-4)		5(4-5)		6(5-6)	
**Mother’s occupation**		0.93		0.24		0.03
Employed	3(2-4)		5(4-5)		6(6-6)	
Homemaker	3(2-4)		4(4-5)		5(5-6)	
**Hearing loss in the family**		0.11		0.57		0.19
One or more individuals	3(2-3)		4 (4-5)		5(4-6)	
Healthy	3(2-4)		4(4-5)		5(5-6)	
**Being monolingual or multilingual**		0.45		0.29		0.1
Monolingual	3(2-4)		4 (4-5)		5(4-6)	
Bilingual	3(2-4)		4(4-5)		6(5-6)	

^a^Mann-Withney U or Kruskal-Wallis Test

**Table 3.** The association Between the Independent Variables Under Study, and the Auditory Performance Scores Obtained 6, 12, and 24 Months After the Implantation (n = 98)

Variable	The Auditory Performance Graph					
	The median of the score in 6 months		The median of the score in 12 months		The median of the score in 24 months	
	Median (q1-q3)	P value^a^	Median (q1-q3)	P value^a^	Median (q1-q3)	P value^a^
**Type of hearing loss**		0.02		0.003		0.001
Hereditary	3(2-3)		4(4-5)		5(4-6)	
Non hereditary	3(2-4)		5(4 -5)		6(6-7)	
**Presence in the rehabilitation program**		0.007		0.001		0.001
Weak	3(2-3)		3(3-4)		4(4-5)	
Average	3(2-3)		4(4- 5)		5(5-6)	
Good	3(2-4)		5(4-5)		6(5-6)	
**Age at the time of implantation, y**		0.1		0.04		0.009
2-4	3(2-4)		5(4-5)		6(5-6)	
Above 4	3(2-3)		4(3-5)		5(4-6)	
**Age difference between the births, y**		0.38		0.52		0.6
Only child	3(2-4)		5(4-5)		5(4-6)	
3 years or lower	3(2-3)		4(3-5)		5(4-6)	
More than 3 years	3(2-4)		5(4-5)		5(4-6)	

^a^Mann-Withney U or Kruskal-Wallis Test

## 5. Discussion

In the present study, the mean of the auditory performance 6, 12, and 24 months after the implantation were obtained as 2.8, 4.36, and 5.34, respectively, which shows the improvement of the children’s auditory performance in the period of 2 years after the operation. In a study which was conducted by Yang in 2004, the auditory performance was measured as 3.93, and 5.86 in 1 and 2 years after the implantation, respectively (12). Moreover, Donoghues conducted a study in 1998, and reported the mean of the auditory performance scores as 4, and 5 in 1, and 2 years after the implantation, respectively (13). Cochlear implantation is accepted as the standard treatment for children with bilateral profound hearing loss who do not respond to other treatments (6). The results of the present study, also, are in line with other studies which show the improvement of the auditory performance in children who have undergone the cochlear implantation (11, 13). In this study, no significant association was found between the age at the time of implantation, and the auditory performance 6 months after the operation. However, this association revealed to be statistically significant 12 and 24 months after the implantation. These findings are in line with the studies conducted by Cohen (14), Mcpherson (15), Tait (16), and a number of other studies (6, 17, 18). Nevertheless, in a study, which was conducted by Olds et al. on the comparison of cochlear implantation between children under 2 years old, and those between 2 and 4 years old, no statistically significant difference was observed between the two groups’ hearing and speaking (19). The results of the present study showed no significant association between sex, and the children’s auditory performance 6, 12, and 24 months after the implantation. Two other variables which were studied in the present research were the mother’s occupation, and level of education which had not been seriously investigated in other studies. Although the auditory performance was expected to improve more in the children whose mothers had a higher level of education, the present study revealed no statistically significant association between the mother’s level of education, and the auditory performance in the 3 stages. This might be due to the limited number of mothers who had higher education in the present study. Furthermore, a significant association was found between the mother’s occupation, and the children’s auditory performance 2 years after the implantation, while this association was not significant 6, and 12 months after the operation. Of course, the limited number of the employed mothers in the present study (8%) might have reduced the accuracy of the study in determining the association between these two variables. The results of a similar research which was performed on German children revealed that the longer the rehabilitation program continues after the operation, the better the performance of the child would be (20). In the same line, Hashemi et al. conducted a study and came to the conclusion that if the rehabilitation program continues for a longer period of time after the operation, children would learn the hearing as well as the speaking skills more effectively (21). The study also depicts a significant association between the length of the presence in rehabilitation program, and the auditory performance scores in children who had undergone the cochlear implantation. The families who let their children take part in the rehabilitation programs for a longer period of time seemed to be more strongly motivated to support them in all aspects. The present study also showed that the association between the number of the references to the rehabilitation program after the operation, and the auditory performance can be statistically significant; therefore, the number of the references to the rehabilitation program must be considered as an influential variable. This variable revealed a significant association with the children’s auditory performance 6, 12, and 24 months after the cochlear implantation. Although the existence of the individuals with hearing loss among one’s close relatives (father, mother, brother, and sister) was expected to have an impact on the auditory performance, and lead one to sign language rather than hearing and speaking abilities, the present study revealed no statistically significant association between these variables and, as a result, was not able to confirm this speculation. Daneshi et al. conducted a study and revealed equal medians for the level of the auditory performance in patients with hereditary and nonhereditary hearing loss. Therefore, no statistically significant difference had been found between the two groups’ level of auditory performance (22). On the other hand, the present study showed a significant association between the type of the hearing loss (hereditary or non-hereditary), and the auditory performance and, consequently, the hearing loss’s being hereditary or not can be of great help in selecting the candidates of the cochlear implantation. Nevertheless, using more precise diagnostic experiments, if genetically possible, can enhance the accuracy of the studies a great deal. Another variable investigated in the present study was the family size which was not seriously taken into account in other studies. This study revealed no significant association between the family size, and the auditory performance scores in the 3 stages. The mean of the family size was quite low in the present study (3.95). 75% of the families had only one or two children, which might have led to the nonsignificant association between these two variables. Also, being monolingual or bilingual did not show any significant association with the auditory performance. Regarding the variety in the ethnicities as well as the accents, identification and classification of the subjects into these two groups was not such an easy task, and could be accompanied by the error of classification. The study which was conducted by Stocks reported the cooperation between the families and the center, the parents’ being conscious, and the child’s learning capability as major factors which accelerate the process of the treatment. The present study also confirmed the highly significant association between the regular participation in the rehabilitation program, and the children’s auditory performance 6, 12, and 24 months after the cochlear implantation. 

In line with other studies conducted on the issue, the present study confirms the improvement of the auditory performance in the children who benefit from the cochlear implantation. Therefore, the society needs to be aware of the successfulness of the cochlear implantation, and trust in this great method of treatment. Moreover, since lower age is considered as the best time for the cochlear implantation, the country’s health policy makers, and health planners must executively take into account the infants’ hearing screening program. Therefore, by timely diagnosis and intervention, the effectiveness of the treatment can be enhanced in the children. In addition, the financial support, and the allocation of more budgets on the part of the managers, and the policy makers as well as the expansion of cochlear implantation center throughout the country can reduce the waiting time for the operation which undesirably increases the children’s age at the time of the implantation.
